# Identifying criminals: No biasing effect of criminal context on recalled threat

**DOI:** 10.3758/s13421-021-01268-w

**Published:** 2022-01-13

**Authors:** Terence J. McElvaney, Magda Osman, Isabelle Mareschal

**Affiliations:** 1 Department of Biological and Experimental Psychology, School of Biological and Chemical Sciences, Queen Mary University of London, London, UK; 2 Centre for Science and Policy, University of Cambridge, Cambridge, UK

**Keywords:** Context effects, Memory, Priming, Perception

## Abstract

To date, it is still unclear whether there is a systematic pattern in the errors made in eyewitness recall and whether certain features of a person are more likely to lead to false identification. Moreover, we also do not know the extent of systematic errors impacting identification of a person from their body rather than solely their face. To address this, based on the contextual model of eyewitness identification (CMEI; Osborne & Davies, 2014, *Applied Cognitive Psychology*, *28*[3], 392–402), we hypothesized that having framed a target as a perpetrator of a violent crime, participants would recall that target person as appearing more like a stereotypical criminal (i.e., more threatening). In three separate experiments, participants were first presented with either no frame, a neutral frame, or a criminal frame (perpetrators of a violent crime) accompanying a target (either a face or body). Participants were then asked to identify the original target from a selection of people that varied in facial threat or body musculature. Contrary to our hypotheses, we found no evidence of bias. However, identification accuracy was highest for the most threatening target bodies high in musculature, as well as bodies paired with detailed neutral contextual information. Overall, these findings suggest that while no systematic bias exists in the recall of criminal bodies, the nature of the body itself and the context in which it is presented can significantly impact identification accuracy.

Eyewitness testimony and perpetrator identification lies at the heart of the criminal justice system. In the absence of incriminating physical evidence, an eyewitness can be crucial in convincing a court of a defendant’s guilt. Despite its importance, a large body of research has found that the capacity of eyewitnesses to identify perpetrators is error prone (Steblay et al., 2001, 2003), and the nature of these errors remains largely unexplored, such that it is unclear if a systematic bias exists in the appearance of those wrongly identified as criminals. Specifically, do their appearances align with that of a stereotypical “criminal”?

Identification errors may be attributable to the difficulty people often experience with recognizing unfamiliar faces. Although early work in facial recognition suggested that people are experts in recognizing unfamiliar faces (Hochberg & Galper, 1967; Yin, 1969), it has since been argued that this “expertise” reflects our capacity to match exact images of people only (Bruce, 1982; Bruce et al., 1999). Even slight tweaks in the viewpoint or lighting of an unfamiliar face can disrupt recognition accuracy (Hancock et al., 2000). Person identification rates in immediate memory tasks have been shown to be as low as 60% correct (Megreya & Burton, 2008), with participants also recognizing the target in target-absent line-ups in 20% of cases.

In addition to difficulty in identifying faces, people may also struggle with accurately recalling other important visual features, such as body morphology. Indeed, body shape plays an important role in our perceptions of others (Aviezer et al., 2008; Aviezer et al., 2012a, 2012b; Bobak et al., 2016b, b; Enea & Iancu, 2016; Meeren et al., 2005; Van den Stock et al., 2007; Vrancken et al., 2017), in how accurately we recall them, particularly in the absence of useful facial information (Rice et al., 2013). However, our capacity for accurate body recall is limited. Buckhout et al. (1974) found that participants were relatively accurate in their estimations of criminal height following a staged mock crime, but underestimated weight by 10–15 kg. Others report the opposite trend, with target weight slightly overestimated and height slightly underestimated (Buckhout, 1980; Buckhout et al., 1975). More recent work (Ebbesen & Rienick, 1998) found that both target height and weight were underestimated, while a study of eyewitness recall under naturalistic settings (Yarmey & Yarmey, 1997) found that estimations for weight and height were only moderately accurate.

In a study on the recall of real-life eyewitnesses who had observed a particular gun-shooting incident, Yuille and Cutshall (1986) found that errors in estimating height, weight, and age accounted for 52% of the person description errors at first recall. This prompted the authors to declare that “witnesses should not be asked to provide descriptive statistics of people. It is obvious that attempts to guess at height, weight, and age are pointless. . . . police should be content with relative estimates of these characteristics. That is, they should ask each witness to make height judgments relative to some environmental fixture, or another individual” (Yuille & Cutshall, 1986, p. 299).

Given that recognition accuracy for unfamiliar faces under controlled laboratory conditions can be low (Bruce, 1982; Bruce et al., 1999; Hancock et al., 2000; Megreya & Burton, 2008), and that body morphology estimations are error prone, it is unsurprising that eyewitness identification is often poor. Indeed, changes to the eyewitness lineup procedure have yielded only relatively small improvements in identification accuracy (McQuiston-Surrett et al., 2006). Lineup procedures typically involve the presentation of one suspect embedded around known innocent distractors, or “foils”, together in a line (simultaneous lineup). A more recent method consists of a witness viewing one lineup member at a time and deciding whether that person matches the appearance of the criminal prior to viewing the next member (sequential lineup; Lindsay & Wells, 1985). This helps to minimize the risk of a witness simply selecting the member who most closely resembles the perpetrator in a relative comparison in a scenario where the actual perpetrator is absent (Steblay et al., 2001).

Despite this potential improvement in lineup procedure, Wells et al. (2015) found that, in a study on actual former eyewitnesses, among those who made an identification, 32% of witnesses in a sequential lineup paradigm selected an innocent foil. Given this, it is unsurprising that eyewitness misidentification of innocent defendants plays a significant role in most cases of prisoners later exonerated through DNA evidence (The Innocence Project, 2022; Wells et al., 1998; West & Meterko, 2016). In actual criminal cases, suspect identification is subject to additional extraneous variables, making the task even more difficult (Wells, 1978). Indeed, many aspects of a crime outside of the control of the justice system (known as *estimator variables*) can affect the accuracy of perpetrator identification. For example, recognition accuracy improves when the physical distance between witness and criminal is small, and the duration the witness views the criminal is long (Lindsay et al., 2008; MacLin et al., 2001; Memon et al., 2003; Pezdek & Blandon-Gitlin, 2005; Wagenaar & Van Der Schrier, 1996).

## Directional biases in misidentification: The CMEI

It is clear that errors can occur in the identification of unfamiliar people accused of crimes; however, it is less clear whether some people are more likely to be erroneously identified as a criminal. The contextual model of eyewitness identification (CMEI; Osborne & Davies, 2014) posits that associating someone with a crime distorts their appearance in later recall by automatically activating a stereotype about a perpetrator’s appearance that is congruent with the crime being committed. This is most frequently illustrated through examples of racial stereotypes, wherein crimes such as identity theft or embezzlement activate a Caucasian stereotype, while crimes such as procuring prostitution and carjacking activate a Black stereotype (Osborne & Davies, 2013). Upon activation, eyewitnesses are primed (Bargh & Chartrand, 2014) to preferentially encode—and later retrieve from memory—features of the perpetrator that are prototypically consistent with that stereotype. As such, the recollection of the criminal will be biased to match a stereotypic culprit more closely. The extent to which the recalled appearance of the perpetrator is distorted by this stereotype is subject to the estimator variables present, with disruptive extraneous factors increasing contextual distortion.

Evidence for the ability of stereotype activation to shape later identification was found by Eberhardt et al. (2003). When participants were presented with an image of a racially ambiguous man, and informed that he was either Black or White, the recalled target was distorted to comply more with the activated racial context. Kleider et al. (2012) report similar findings, showing that participants misidentified people with stereotypically Black features (as rated subjectively by a set of participants) as drug dealers significantly more than those with less stereotypical features. In a direct test of the CMEI, Osborne and Davies (2013) demonstrated that framing a target as the perpetrator of a stereotypically “Black” crime (drive-by shooting) resulted in participants misidentifying the target as significantly more stereotypically Black than when framed as the perpetrator of a stereotypically “White” crime (serial killer). Furthermore, Ben-Zeev et al. (2014) found that when participants studying a Black face were subliminally primed with a word that was counterstereotypic of Black people, they subsequently recalled the face as Whiter than participants primed with a stereotypic “Black” prime. These findings are consistent with the CMEI’s prediction that context can result in witnesses recalling a face as more racially congruent with the provided information.

## The CMEI, perceived threat, and criminality

According to the CMEI, criminal stereotype activation is not restricted to race. For example, the authors argue that prostitutes are associated with relatively feminine features (Ward et al., 2012), and therefore suspected prostitutes are more likely to be misidentified if they are high in perceived female gender stereotypicality (i.e., if they have relatively feminine features). Similarly, when someone is associated with a criminal act (Flowe, 2012), they argue that they should later be recalled as appearing less trustworthy than when they are associated with a neutral act. There is some support for this nonracial memory biasing. For example, in a facial recreation study, participants recalled an identical face as less intelligent and attractive (as rated by a separate set of participants) when they were told it depicted a murderer as opposed to a lifeboat captain (Shepherd et al., 1978). In a rare study on the biased recall of body morphology (Shaw & Wafler, 2016), participants were presented with footage of a staged crime and then asked to select the culprit from a (suspect-absent) lineup that consisted of digitally manipulated defendants of varying body morphologies. Suspects who were altered to appear muscular were significantly more likely to be identified as the criminal than suspects who more closely matched the morphology of the actual perpetrator. These results suggest that, as per the CMEI prediction, the suspect was perceived and later recalled in a biased fashion to match the appearance of a stereotypically threatening criminal. Indeed, perceived threat in computer-generated (CG) body stimuli has previously been shown to increase linearly with increases in musculature (McElvaney et al., 2021).

However, this prediction that a criminal framing can subsequently modify the manner in which appearance is processed and recalled has yet to be systematically explored. One key attribute associated with a “criminal appearance” is how threatening a person appears. In a study on the perception of criminality, Funk et al. (2017) found that the perceived threat of a face was highly positively correlated with the criminality associated with the face. In addition, Oosterhof and Todorov (2008) found that high perceived threat is a result of a combination of high dominance and low trustworthiness, which has been shown to be associated with harsher, more extreme criminal sentencing decisions (Wilson & Rule, 2015, 2016). Hence, the perception of a stereotypical criminal is likely to be biased towards high threat. Thus, if a person is associated with committing a crime, this should distort their recall by participants to align with that inherent stereotype (i.e., appearing more threatening; MacRae et al., 2002).

Research on the effects of a positive or negative framing context on person identification has yielded inconsistent results. Recognition accuracy for people paired with prosocial contexts is higher than when paired with antisocial or neutral contexts (Felisberti & McDermott, 2013; Felisberti & Pavey, 2010). Moreover, informational contexts that frame the target as belonging to an out-group of a lower social class than the participant result in something like a cross-race effect (CRE; Sporer, 2001), wherein recognition is impaired for targets outside of the participant’s cultural or socioeconomic/class circle (Marsh, 2021; Shriver et al., 2007). These observations broadly align with the CMEI model, whereby the encoding and memory of people with unsavoury behaviours should be biased or impaired.

However, the opposite pattern has also been found. For example, face identification is enhanced when people are framed as having a history of cheating (Mealey et al., 1996). Similarly, neutral male faces depicted as defectors in the Prisoner’s Dilemma game are identified with greater accuracy than cooperating males (Oda, 1997). Simply pairing faces with pleasant, neutral, or unpleasant information can impact later identification, with some studies showing that faces presented in unpleasant contexts are recalled with greater accuracy than positively/neutrally valanced faces (Mattarozzi et al., 2015; Mattarozzi et al., 2019). Faces that inherently appear untrustworthy are often recalled more accurately that those with trustworthy appearances (Rule et al., 2012).

These results run counter to the predictions of the CMEI and suggest that recognition for appearance may be enhanced by the presence of negative character trait information, or affectively salient contextual information (Kerr & Winograd, 1982). However, none of the above studies directly assessed the nature of the people misidentified in the wake of being initially framed in a threatening context. The incorrect choice alternatives (foils) were not varied systematically in their perceived threat relative to the target, making it difficult to conclude whether the targets were actually encoded in a biased fashion.

## The current study

If the appearance of those accused of a crime is encoded in such a way that they are recalled as more threatening, this suggests a potential bias against people with naturally more threatening appearances. Hence, the goal of the current study was to conduct a systematic examination of the contextual effects of crime information on the identification of criminal faces and bodies.

We measured the impact of context using still images of CG face stimuli varying in perceived facial threat (Todorov et al., 2013) and CG body stimuli varying in musculature. We asked participants to study the image and later identify it from the foils. Although this does not match the procedural experience of real eyewitnesses, this allowed us to explore the potentially biasing effects of criminal context while maintaining tight control over the stimuli. We explored this potential bias to recall targets as more threatening when framed in criminal contexts in three experiments using (i) an immediate memory paradigm with a simultaneous lineup of targets and foils, (ii) a delayed memory paradigm with a sequential lineup of targets and foils, and (iii) a delayed memory paradigm with a simultaneous lineup of foils only. We hypothesized that, in the absence of criminal context, participants would not show any particular directional bias in recalling a body or face. We also hypothesized that, consistent with the CMEI, participants would select more threatening faces/larger bodies when the original target was presented in a criminal context than when presented in a neutral context. Each experiment and all hypotheses were preregistered before data collection began—Experiment 1 (https://osf.io/a82rw/), Experiment 2 (https://osf.io/4pmc5/), and Experiment 3 (https://osf.io/he84j). Ethical approval was granted by the Queen Mary University of London Institutional Review Board.

## Experiment 1

We examined the effect of providing a context vignette (“framing”) of target CG faces and bodies as perpetrators of crimes on the encoding and later identification of these targets in an immediate memory paradigm. Participants were briefly presented with a target face or body, and then asked to identify the target from a lineup of either similar faces varying in perceived threat or similar bodies varying in musculature . Half of the participants were given a contextual backstory depicting the targets as perpetrators of a violent crime and informed their task would be to identify the targets, while the other half were given no contextual information (no context).

We first hypothesized (H1) that, in the no-context condition, participants would not be systematically biased in selecting a body or face. Second (H2), we predicted that participants would select more threatening faces/larger bodies in the criminal-context condition than in the no-context condition. We further expected an interaction between condition and the threat/musculature level of the target presented. Namely (H3), we expected the effect of the criminal context to be stronger for less threatening/smaller stimuli for two reasons. First, previous studies have shown that, when presented with a subliminal prime that contradicts the stereotypical nature of a presented stimulus, participants recall the stimulus as more closely matching the prime (Ben-Zeev et al., 2014). For the current experiment, if a nonthreatening/smaller person was accused of a crime, participants may subsequently select a stimulus that aligns more with the criminal context, i.e., a more threatening/larger person. Second, given the nature of the stimuli, smaller targets had a greater number of larger foil (more threatening) options available than larger targets. Hence, if the effect of crime condition emerged, it would be more pronounced in the less threatening/smaller stimuli.

### Methodology

#### Participants

To determine the required sample size for the current experiment, we conducted a power analysis using G*Power3 (Faul et al., 2007). For a mixed-methods design (ANOVA: Repeated measures, between factors), assuming a small-medium effect size (Cohen’s *f* = 0.20) a total sample size of 220 participants was required to assess the effects of both target stimulus (three levels of measurement) and context (two groups) on selection accuracy with a power of .95 and alpha level of .05. The experiment was conducted in April 2020 via the online software Qualtrics, with participants recruited from Prolific, an online crowdsourcing platform, and paid £0.85 for taking part. Participants were located in the UK, over 18 years of age, fluent English speakers, and had achieved an approval rate of at least 85% in their previous Prolific study participations (61 males; age: *M =*34.96 years, *SD =*12.57).

#### Stimuli (faces)

**Fig. 1:**
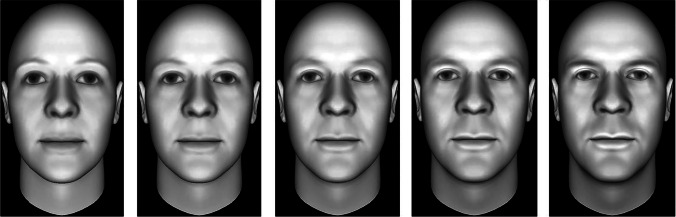
Five levels of facial threat in one identity face. Left-to-right: −2SD, −1SD, 0SD, +1SD, +2SD, from Todorov et al. (2013)

We selected 10 different identity face stimuli from the Todorov et al. (2013) dataset of computer-generated faces that have been previously validated to vary along the dimension of threat. All faces in the dataset are Caucasian male, and we selected those that appeared the least ambiguously Caucasian and also the least feminine in the low threat range. We converted the stimuli to greyscale and selected one face from each of these 10 sets of identity faces to act as the target stimulus. Six of these targets were at the central level of threat, 0SD (see Fig. 1), while the other four targets consisted of two faces each at −3SD and +3SD to avoid expectation effects.

#### Stimuli (bodies)

The body stimuli were realistic CG human male figures created using Daz Studio 4.10 Pro (https://www.daz3d.com/daz_studio) with the Male Anatomy Smart Content package. This software provides a default, anatomically accurate standard model (‘Genesis 8 Basic Male’) with dimensions that can be modified precisely to allow fine control over individual body shapes.

**Fig. 2:**
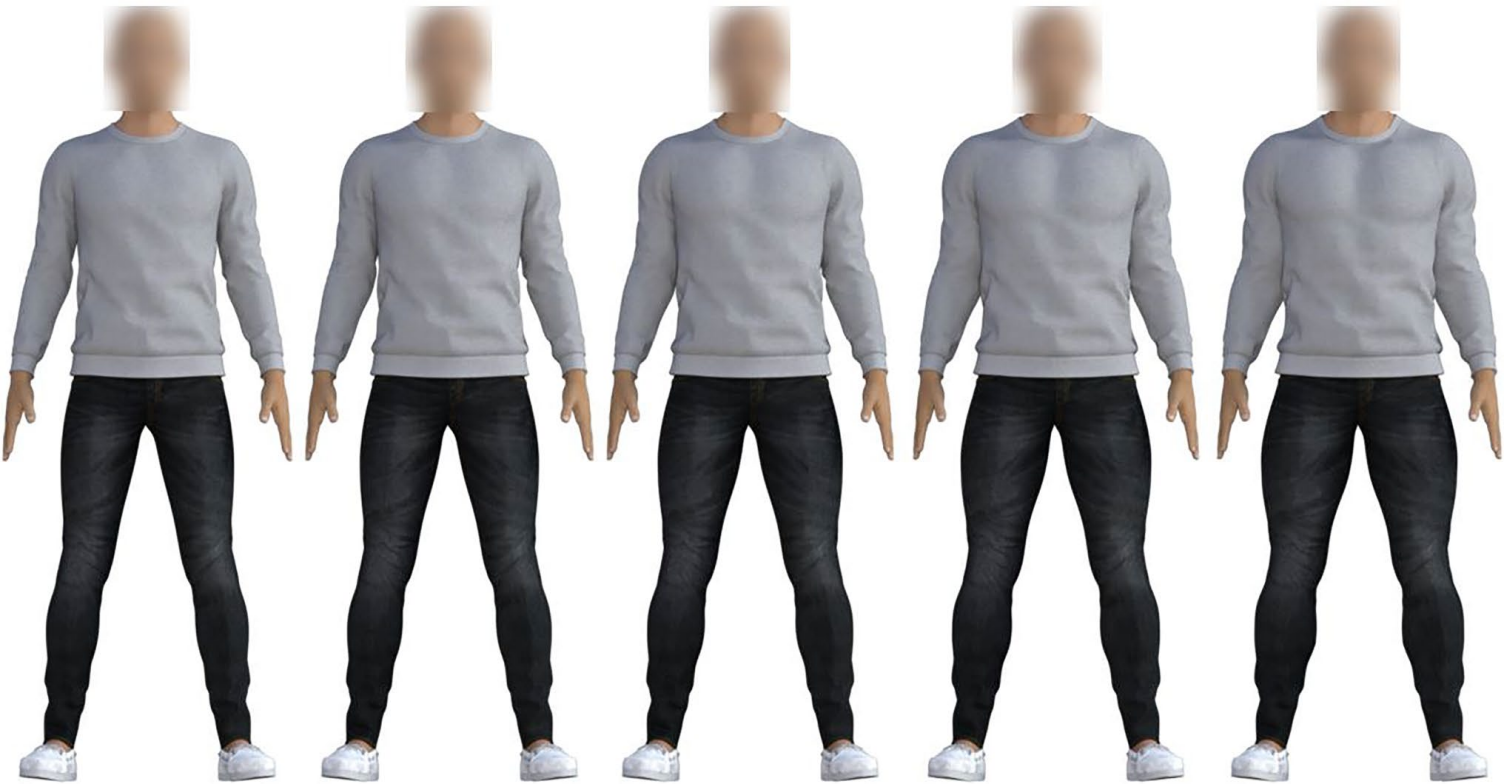
Various levels of musculature in Experiment 1. Left-to-right: 0%, 25%, 50%, 75%, 100%. Target stimulus (e.g., 50% musculature) shown in the centre

We manipulated the body’s appearance using the in-built musculature scale on the initial standard model to create target stimuli at three levels of musculature: 0% (unaltered), 50%, and 100%. There were 10 target identity bodies (using different clothing and blurred faces), six targets were at the central level of musculature (50%), two were at 0%, and two were at 100% musculature to avoid expectation effects. Foils of each target were created using step intervals of 25% musculature (see Fig. 2; see Appendix 1 for details).

#### Procedure

Participants were randomized into either a criminal-context (*N* = 110) or no-context (*N* = 110) condition. In the criminal-context condition, they were informed that they were to adopt the role of an eyewitness, and that they would be presented with CG recreations of people accused of a crime. Participants were informed that the person they were about to see was accused of manslaughter, having shot and killed an innocent bystander while committing a robbery. In the no-context condition, participants were informed that the goal of the experiment was to examine how accurately they could identify unfamiliar people after a brief presentation.

**Fig. 3:**
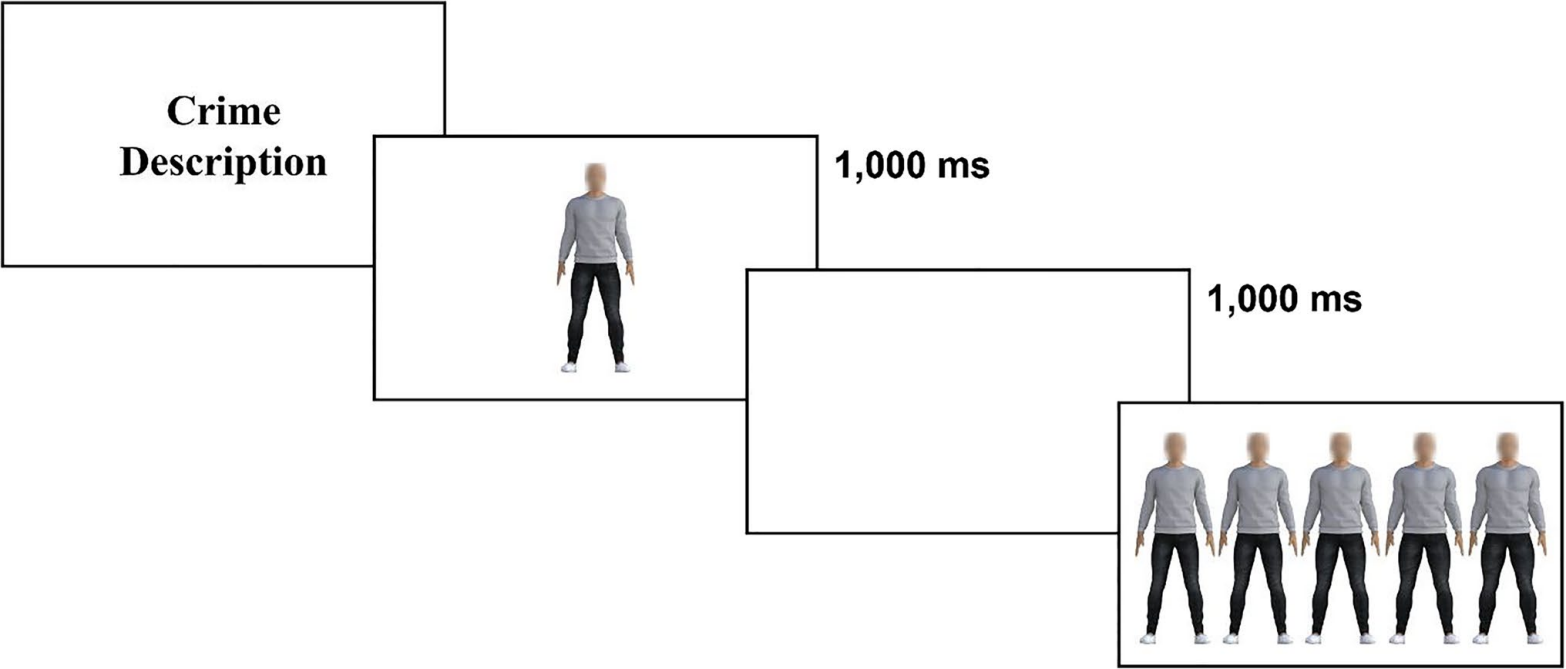
Experiment 1 sample body trial in criminal-context condition. Participants read the crime of which the target was accused. This was followed by a brief presentation of the target (1,000 ms), followed by a blank screen (1,000 ms). Participants then selected the target from a lineup of five possibilities (no time limit imposed)

Participants completed 20 trials in total, 10 target face trials (six trials at 0SD, two trials at −3SD, and two trials at +3SD) and 10 target body trials (six trials at 50% morphology, two trials at 0%, and two trials at 100%), using a different identity body/face target in each trial. In the criminal-context condition they were provided the same crime information prior to each trial, and then presented with the target stimulus (1 second), followed by a blank screen (1 second), followed by the lineup. In the no-context condition there was no information prior to each trial, and they were presented with a target stimulus on screen (1 second), followed by a blank screen (1 second), followed by the lineup (see Fig. 3). Body and face trials were randomly interleaved, and the participants’ task was to select the target from the lineup. After confirming their selection, participants proceeded to the next face or body trial.

For face-target conditions, there were five options on the screen (the target and four incorrect foils randomly positioned on each trial) of the same identity face varying in threat. The options stayed on the screen until the participant made their selection (see Fig. 3). For the 0SD target-face conditions, the five options consisted of faces at −2SD, −1SD, 0SD, +1SD, and +2SD. For the −3SD target face condition, the five options consisted of faces at −3SD, −2SD, −1SD, 0SD, and +1SD. For the +3SD target-face condition, the five options consisted of faces at +3SD, +2SD, +1SD, 0SD and −1SD.

For body-target conditions, participants were presented with five same identity body options on screen in a randomized order. One of these was the original target body stimulus and the others were foils. On all trials, the five options consisted of the identity body at five levels of musculature: 0%, 25%, 50%, 75%, and 100%.

To check that increased musculature increased perceived threat, at the end of the experiment, participants were randomly presented with five new body stimuli wearing the same clothes and varying in musculature and asked to rate the perceived threat of the bodies on a scale of 1 to 7.

### Results

#### Musculature check

We used a mixed-effects ordered logit to assess the influence of level of musculature on perceived threat (PT) in the body stimuli. The overall model was significant in predicting the variation in PT of the body stimuli, Wald χ^2^(1) = 270.19, *p* < .001. Consistent with previous research (McElvaney et al., 2021), we found a significant stepwise, linear impact of musculature, with each increment accompanied by an increase in PT, *OR* = 3.74, 95% CI [3.19, 4.37], *p* < .001.

#### Descriptive statistics

Mean accuracy (proportion of correct identifications) was found to be higher for faces (*M* = .57, *SE* = .01) than for bodies (*M* = .49, *SE* = .01). In both cases accuracy was lower than the accuracy in a pilot study (Pilot 2) where participants made no errors when they simply had to match the target stimulus to the correct option (with target still on screen). For faces and bodies in our task, accuracy was also still well above chance (0.2). No clear difference emerged in performance between those in the criminal-context condition (*M* = .54, *SE* = .01) and those in the no-context condition (*M* = .52, *SE* = .01). A breakdown of performance across the two conditions can be found in Table 1.

**Table 1:** Identification accuracy (proportion of correct responses correct)

			Mean	*SE*	95% CI
Face Acc.			.57	.01	[.55, .60]
Body Acc.			.49	.01	[.46, .51]
Crime Acc.			.54	.01	[.52, .57]
No Context Acc.			.52	.01	[.49, .55]
Facial Threat					
	−3SD				
		Crime	.62	.03	[.56, .69]
		No Context	.60	.03	[.53, .66]
	0SD				
		Crime	.43	.02	[.39, .47]
		No Context	.44	.02	[.41, .48]
	+3SD				
		Crime	.70	.03	[.63, .77]
		No Context	.65	.04	[.57, .72]
Body Musculature					
	0%				
		Crime	.52	.04	[.45, .59]
		No Context	.48	.04	[.41, .55]
	50%				
		Crime	.37	.02	[.33, .41]
		No Context	.39	.02	[.35, .43]
	100%				
		Crime	.62	.04	[.55, .69]
		No Context	.55	.04	[.48, .62]

#### Directional bias in central stimuli

##### No context

To assess directional biases in participants’ responses, we focused on no context trials using the central target stimulus (0SD Threat Face or 50% Musculature) that had equal probability of choices above or below the target (face/musculature). If the correct face/body was selected, this response was coded as a difference score of zero. If the participant overestimated (underestimated) the threat/muscle level by one or two levels, this difference was coded as a score of +1/+2 (−1/−2) respectively. To investigate our first hypothesis, we used one-sample *t* tests to assess whether the mean difference scores for the no context central stimuli deviated significantly from the correct target value of zero. Consistent with H1, for the bodies, we found that the mean selected body (*M =* 0.04, *SE =* 0.03) did not vary significantly from the target body, *t*(109) = .336, *p* = .738, *d* = .03. However, for the faces, the mean selected face (*M =* 0.09, *SE =* 0.04) was significantly higher in threat than the target face, *t*(109) = 2.37, *p =* .019, *d =* .22.

#### Bias across all stimuli

To test our second and third hypotheses, we calculated absolute difference scores for each level of facial threat and musculature. We then used mixed ANOVAs to test the influence of target facial threat/musculature (within-participant) and the presence of criminal context (between-participant) on absolute difference scores.^1^ As the absolute difference score for the noncentral trials could vary from 0 to 4 (since foils could be a maximum of four steps away), while the potential absolute difference for the central target trials varied from 0 to 2 (since foils could be a maximum of two steps away), we did not analyze the within-participant difference between the central stimuli and extreme stimuli.

**Fig. 4:**
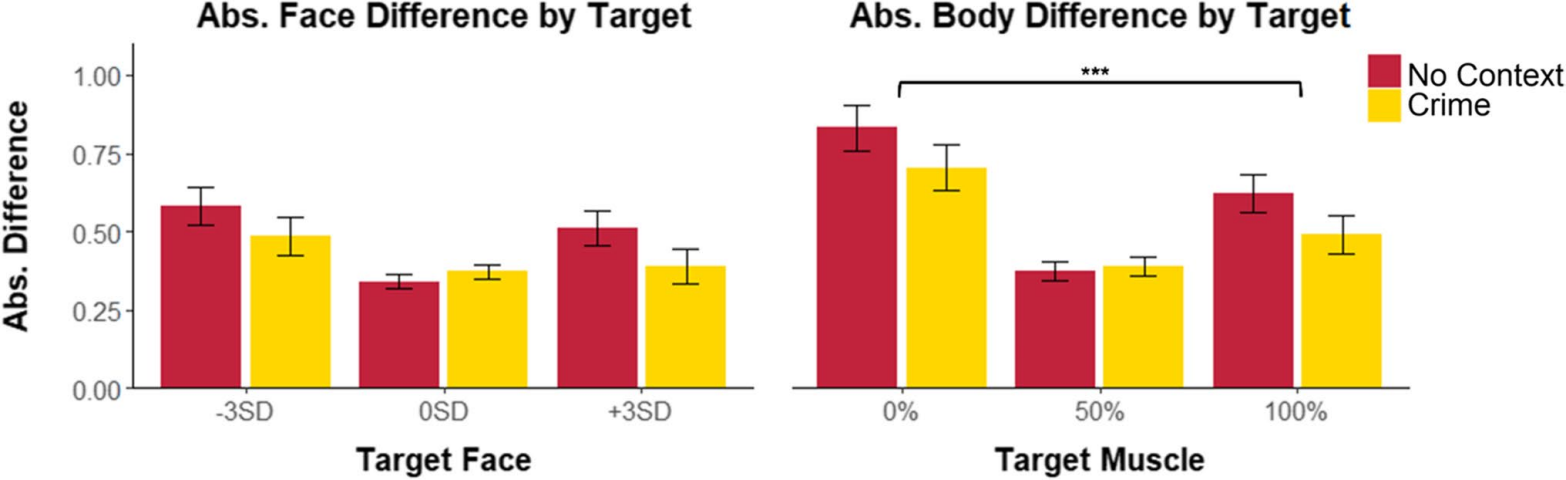
Plots of mean absolute difference of selected face from target face (left) and selected body from target body (right). Error bars represent ±1 *SE*

We found no effect of target facial threat or criminal context on absolute difference scores for the facial trials, nor any significant interaction. Similarly, an exploratory analysis of response accuracy (correctly identifying the target stimulus) to facial trials revealed no effect of target threat or context. We did find a main effect of musculature on absolute difference scores, *F*(1.72, 374.05) = 24.08, *p* < .001, partial η^2^ = .10. The absolute distance of the mean chosen body from the target was significantly higher for the 0% muscle targets (*M =*.77, *SE =*.05) than for the 100% muscle targets (*M =* .51, *SE =* .04), *p =* .004, *d* = .22 indicating better performance on the larger bodies (Fig. 4).

Similarly, an exploratory analysis of response accuracy to body trials revealed a significant effect of musculature, *F*(1.85, 403.38) = 20.39, *p* < .001, partial η^2^ = .09, with accuracy for target selection significantly higher for the 100% muscle targets (*M =* .58, *SE =* .03) than for the 0% muscle targets (*M =* .50, *SE =* .03), *p =* .020, *d* = .15. Contrary to H2 and H3, we found no effect of context, nor any interaction.

#### Experiment 1 discussion

Contrary to our predictions, we found that pairing faces and bodies with criminally charged contextual information had no effect on the recall of either the face or body. The lack of significant differences between groups is unlikely to be due to ceiling/floor effects. Indeed, if contextual information had biased recall on the faces or bodies, we would have expected accuracy to go *down* relative to the no context case, which was not the case. Rather, according to the CMEI, the activation of a biasing stereotype is contingent on the moderating influence of estimator variables and system variables (Osborne & Davies, 2014). One key estimator variable thought to impinge on successful eyewitness identification is delay, or the length of time between witnessing the crime and later attempting to identify the perpetrator (Behrman & Davey, 2001; Deffenbacher et al., 2008). Due to the use of an immediate memory paradigm in the current experiment, it is possible that such a distortion may not have had sufficient time to manifest. Furthermore, it is possible that employing a longer, more detailed crime vignette (like that used by Yang et al., 2019), along with a reduction in the number of target faces/bodies presented might increase the salience of the criminal context.

## Experiment 2

To address these issues, we conducted a second experiment, broadly following the methodology of Ben-Zeev et al. (2014) and included a neutral-context condition so participants had a text to read in one of the control conditions. There were three different conditions: (a) description of a crime, (b) description of a neutral event, and (c) no context. Participants studied a target face/body. To add delay, they then completed a distractor task, followed by with an individually viewed face/body trial, where participants indicated whether the stimulus was identical to the target (yes/no task). Participants also rated their confidence in their response (see Appendix 2 for confidence measures results).

We hypothesized that performance in all 3 conditions would be better on easier foil trials (foils further/more distinct from the target) than on difficult foil trials (foils closer to the target) (H1). Second, as person information has been shown to facilitate facial memory, we predicted that participants would make more errors (overall) in the no-context condition than in the neutral condition (H2). Third, we expected that participants in the crime condition would make more errors of identification on the target trials than in the neutral condition (H3). Fourth, we predicted that participants in the crime condition only would show a directional bias in their responses, making more errors of identification for more threatening/muscular face/body foils than for less threatening/muscular face/body foils (H4). Crucially, we expected an interaction between context condition and threat/musculature, such that participants in the criminal-context condition would make more errors of identification on more threatening foil trials and less errors of identification on less threatening foil trials than those in the neutral context and no-context conditions (H5). Furthermore, we expected participants in the criminal-context condition to make more errors of identification on the most threatening foil trials and fewer errors of identification on the least threatening foil trials than those in the other two groups (H6).

### Methodology

#### Participants

For a mixed-methods design (ANOVA: repeated measures, between factors), assuming a small-medium effect size (Cohen’s *f* = 0.22), our power analysis indicated that a minimum sample size of 192 participants would be required to assess the effects of both context condition (three groups) and level of threat/musculature (six levels of musculature) on participant response accuracy with a power of .95 and alpha level of .05. For the current study, this was increased to 200. Participants were recruited in July 2020 from Prolific (52 males; age: *M* = 32.34 years, *SD* = 11.04) and paid £1.50 for taking part. The experiment was built in JavaScript using PsychoJS, an online variant of PsychoPy (Peirce et al., 2019) and hosted on Pavlovia.org.

#### Stimuli

##### Faces

We selected one identity stimulus (seven levels of threat) from Todorov et al. (2013) and converted the faces to greyscale. The neutral threat face (0SD) was the target stimulus while the remaining six variants of this identity face (−3, −2, −1, +1, +2, and +3SD) were foils.

##### Bodies

The body stimuli were realistic CG human male figures, created using Daz Studio, whose faces were blurred and wearing identical clothing. Because only one body stimulus was presented on-screen at a time, this allowed us to use smaller step differences in musculature of 16.67%, resulting in stimuli that varied in seven levels of test musculature; 0%, 16.67%, 33.33%, 50%, 66.67%, 83.33%, 100% (henceforth referred to as Musculature Levels 1–7). We selected the central body (50% musculature/Level 4) to be the target body stimulus and the remaining six variants to be the foils.

##### Vignettes

We designed two crime vignettes based on those originally used in studies by Porter et al. (2010) and Mendelsohn and Sewell (2004; see Appendix 3). Crime Vignette 1 described a shop robbery resulting in a murder, while Crime Vignette 2 described a street mugging also resulting in a murder. We then created two neutral vignettes that matched the crime vignettes in length and setting. Neutral Vignette 1 described someone purchasing a winning lottery in a shop, while Neutral Vignette 2 described someone asking for directions on a street corner. In all conditions, one vignette was randomly allocated to the face stimuli and the other to the body stimuli

#### Procedure

Participants were randomized into one of the three context conditions. In the crime and neutral conditions, they were presented with one of the vignettes and told it described actions by a person about to be presented on screen that they would need to identify later (no time limit on reading was imposed). Participants in the no-context condition were simply told to study the person on the screen for later identification. In all conditions, they were given 30 seconds to study the stimulus. To introduce a delay before the identification stage of the experiment, they then completed eight trials of a modified *n*-back distractor task where they were presented with a random sequence of numbers and, at random intervals, asked to input the previous three numbers from the sequence. This distractor task lasted for approximately 5 minutes. Following this, participants did the identification task in a block of 24 trials, using either a body-target stimulus or a face-target stimulus in a counterbalanced order across participants.

In a replication of the procedure of Ben-Zeev et al. (2014), on a given trial the presented stimulus was randomly selected to be either the (central value) target or one of five valued foils. Target and foils were randomly repeated four times resulting in 24 trials. When participants completed one block of 24 trials for one stimulus type (e.g., body), they were given a second context vignette (or none in the no-context condition) and repeated the same procedure with the other stimulus type (e.g., face).

**Fig. 5:**
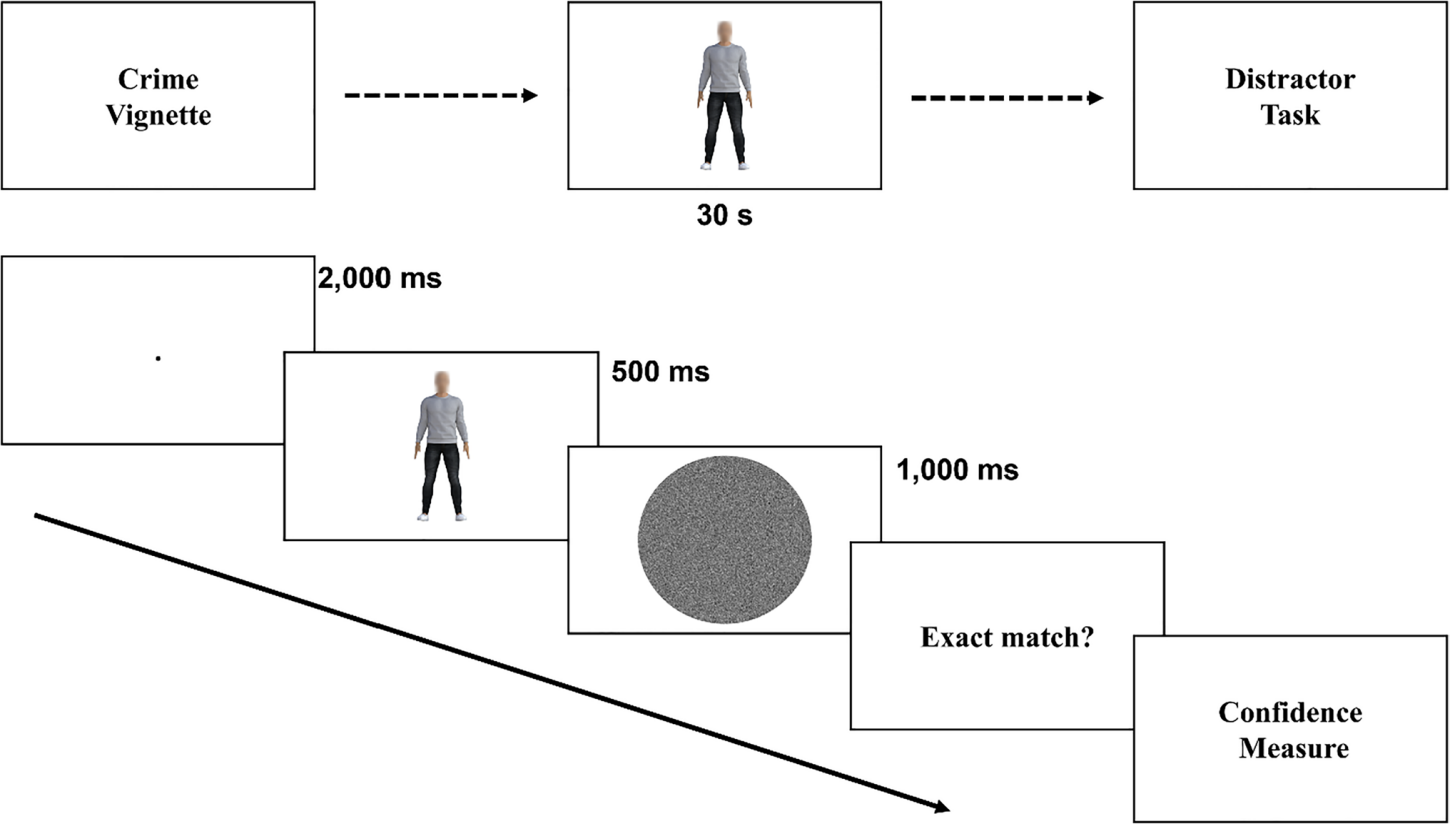
Experiment 2 sample body trial in criminal-context condition. Participants read a detailed crime vignette, then studied the target stimulus for 30 seconds, followed by a distractor task (5 minutes). Each trial consisted of a fixation dot (2,000 ms), the presentation of a foil/target stimulus (500 ms), followed by a random noise mask (1,000 ms). Participants were then asked whether the presented stimulus matched the original target, and to rate their confidence in the decision. Participants completed 24 trials for each face/body target stimulus

In each block, a trial started with a fixation dot presented for 2,000 ms, followed by a stimulus for 500 ms, followed by a random noise mask for 1,000 ms. After the mask was extinguished, participants saw a response screen that asked them to answer Yes/No (using a key press) to the question “Did that face/body EXACTLY match the one you previously studied?” and then rate, on a scale from 1 to 100, how confident they were in their response (see Fig. 5 and Appendix 2 for confidence ratings).

To counteract a potential regression to the mean where participants may have recognized that the true target stimulus lay at the centre of the threat/musculature of the stimuli, each participant was only presented with foil trials consisting of five out of the six possible foil levels, randomly excluding either the most/least threatening face trials and most/least muscular body trials.

### Results

#### Face accuracy

**Fig. 6:**
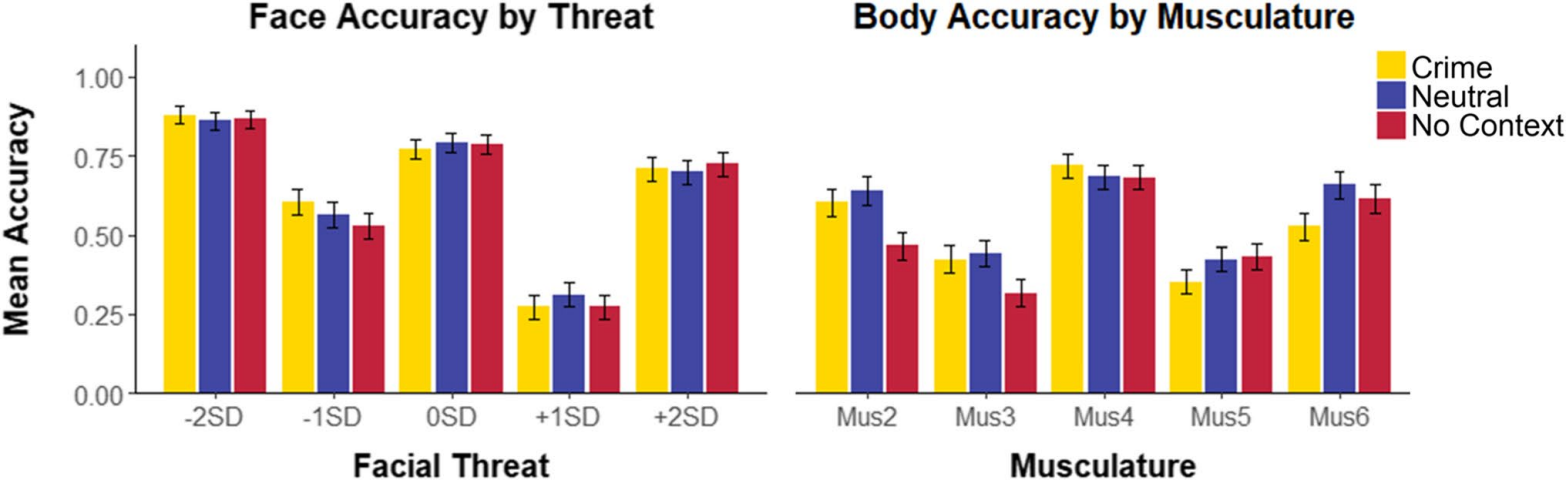
Mean response accuracy for face (left) and body (right) in Experiment 2. Error bars represent ±1 *SE*

We examined accuracy (proportion of correct responses, including hits and correct rejections) across the varying levels of test facial threat (−2SD, −1SD, 0SD, +1SD, and +2SD) between the three context conditions. Since participants were only exposed to one of the extreme face foils (−3SD/+3SD), responses to these extreme trials were analyzed separately. Accuracy was calculated for each threat level by averaging the participant’s performance across each of the four trials completed at that level (Fig. 6). We used mixed ANOVAs to assess the effects of context (between-subject) and test threat level (within-subject) on participant accuracy. Consistent with H1, we found a significant effect of test threat level on accuracy, *F*(2.92, 574.81) = 123.88, *p* < .001, partial η^2^ = .386. Bonferroni corrected pairwise comparisons indicated significant differences (*p* < .05) in accuracy between all threat levels. Accuracy was highest on the least threatening −2SD trials (*M* = .87, *SE* = .02), followed by the 0SD trials (*M* = .78, *SE* = .02), followed by +2SD trials (*M* = .71, *SE* = .02), −1SD trials (*M* = .57, *SE* = .02) and +1SD trials (*M* = .29, *SE* = .02). Contrary to H2–H4, we found no main effect of context nor any interaction between context and threat level.

Finally, we conducted a two-way between groups ANOVA to examine the effects of context condition and facial threat on performance on the most/least threatening face trials (−3SD/+3SD). Again, we found a significant effect of facial threat, *F*(1, 194) = 7.66, *p* = .006, partial η^2^ = .038, with performance significantly higher on the least threatening trials (*M* = .97, *SE* = .02) than on the more threatening trials (*M* = .90, *SE* = .02). However, no main effect of context condition was found and, contrary to H5, no interaction between facial threat and context was seen.

#### Body accuracy

We examined accuracy on the body trials across the varying levels of musculature (Levels 2–6) and between the three context conditions. Responses to extreme body foils (Level 1/Level 7) were analyzed separately. Consistent with H1, we found a significant effect of musculature level on accuracy, *F*(2.70, 531.88) = 29.10, *p* < .001, partial η^2^ = .129, with highest accuracy when the stimulus test was at the target level (Level 4) (*M* = .70, *SE* = .02). Bonferroni corrected pairwise comparisons revealed significantly higher (*p* < .05) performance on the target level test trials than all other stimuli test levels except Level 6 (*M* = .60, *SE* = .03). Performance on Level 6 was higher than all other trials other than Level 2 (*M* = .57, *SE* = .03). Accuracy on Level 2 was significantly higher than that on Levels 3 (*M* = .40, *SE* = .02) and Level 5 (*M* = .40, *SE* = .02).

Moreover, we found a main effect of context, *F*(2, 197) = 4.42, *p* = .013, partial η^2^ = .043. In line with H2, performance in the neutral context condition (*M* = .57, *SE* = .02) was significantly higher than that in the no-context condition (*M* = .50, *SE* = .02), *p* = .012, *d* = .58. There was no significant interaction between context condition and musculature. Contrary to H3–H5, participants did not make more errors in the crime condition than the neutral condition on the target trials or on foil trials higher in musculature than the target.

Finally, a two-way between-groups ANOVA was conducted to examine the effects of context condition and musculature on performance on the most/least muscular body trials (Level 1/Level 7). However, we found no effects of context or musculature, nor a significant interaction.

### Experiment 2 discussion

Consistent with the results of Experiment 1, we found that pairing targets (faces or bodies) with criminally charged contexts did not significantly bias participant recall towards larger/more threatening foils. Identification accuracy was similar in the crime condition to that in the neutral information condition, despite the more detailed crime vignette, and a crime description that only related to a single target rather than different targets.

A possible explanation is that we included target-present trials. It is possible that many cases of false eyewitness identification arise under conditions where the actual criminal is not present in the lineup. According to the CMEI, innocent people whose appearance more closely matches that of a stereotypical criminal will be more likely to be misidentified. For example, Shaw and Wafler (2016) found that participants who witnessed staged crimes were more likely to misidentify more muscular defendants as criminals in the absence of the target criminal. However, their study lacked nuanced control over the body muscularity of the defendant stimuli and included facial information. Furthermore, it lacked a no-crime condition, instead using two different types of crime scenario. Thus, it is difficult to conclude that it was the actual criminal context that drove their pattern of results as opposed to a general tendency to recall bodies as more muscular.

## Experiment 3

We conducted a final experiment aimed at assessing the potential bias in target-absent lineups, using body stimuli only. As in Experiment 2, participants were presented with a target body linked with either a criminal context, a neutral context, or no context at all. Following a distractor task, they were asked to identify the original target from a selection consisting of lower and higher musculature foils only, presented together in a randomized lineup on screen.

As person information has been shown to facilitate facial memory, we predicted that participants would make smaller errors (choose bodies that more closely match the original target) in the neutral condition than in the no-context condition (H1). We also predicted that participants in the crime condition only would show a directional bias in their responses and select more muscular body foils than the target (H2). Finally, we expected that participants in the crime condition would significantly differ from the neutral and no-context conditions in the nature of the foil selected, such that they would select significantly more muscular body foils (H3).

### Methodology

#### Participants

Our power analysis indicated that, for a one-way between-groups ANOVA (ANOVA: fixed effects, omnibus, one-way), with a between-groups factor of 3 levels, assuming a medium effect size (Cohen’s *f* = .25) a minimum sample size of 252 participants would be required to assess the effects of context condition on participant foil selection with a power of .95 and alpha level of .05.

Participants (69 males, 183 females; age: *M* = 26.85 years, *SD* = 11.75) were recruited in January 2021. The sample consisted of a mixture of undergraduate psychology students who were awarded one course credit for taking part in the study (*N* = 95), wth the remainder (*N* = 157) recruited from Prolific and paid £0.90 for taking part. The experiment was built and hosted on Gorilla Experiment Builder (www.gorilla.sc; Anwyl-Irvine et al., 2019).

#### Stimuli (bodies)

The body stimuli were realistic CG human male figures created using Daz Studio, with appearance varying in levels of musculature. The target body stimulus was at the central point of the musculature scale (50%). Since the target stimulus was never tested at identification stage, we used foils that were closer to the target on the musculature scale, covering 12.5%, 37.5%, 62.5% and 87.5% musculature. The default faces on the bodies were blurred and each was dressed in identical clothing, as in Experiments 1 and 2.

#### Procedure

**Fig. 7:**
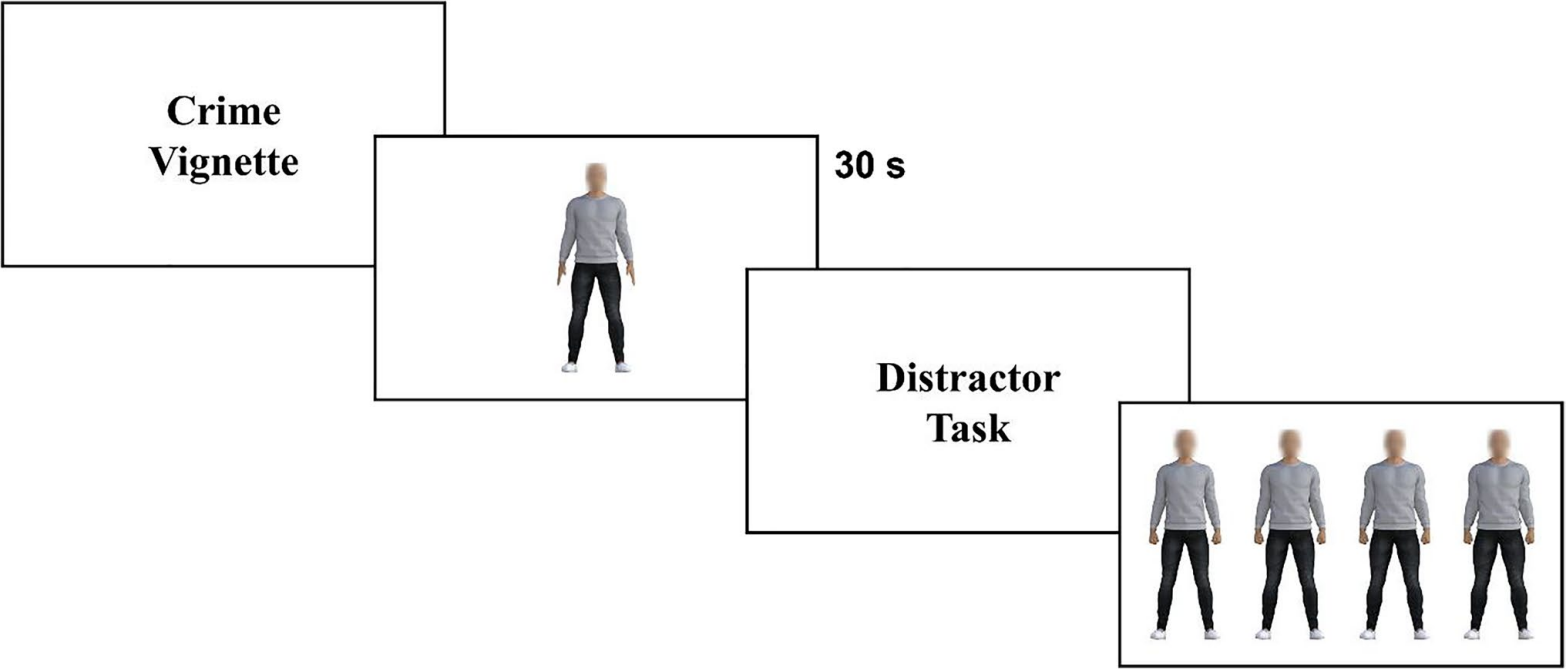
Experiment 3 procedure (criminal-context condition). Participants read a detailed crime vignette, then studied the target body stimulus (30 seconds), followed by a distractor task. Participants were then presented with a lineup of four foils and asked to select the one that matched the target

Participants were randomized into one of three context conditions. In the crime and neutral conditions, they were presented with either Crime Vignette 2/Neutral Vignette 2 (as used in Experiment 2; see Appendix 3) and told it applied to the action of a person about to be presented on screen. There was no time limit on reading the vignette. They were then presented with the body target stimulus on screen for 30 seconds and asked to study it for later identification. To introduce a larger delay, participants completed a modified *n*-back task before the identification task which lasted approximately 10 minutes. The identification task consisted of a single trial, where they were presented with the four body foils in a randomized-order lineup on the screen and asked to identify the one matching the previously viewed target (Fig 7). Crucially, none of these bodies matched the original target, as this was a target-absent paradigm.

### Results

**Fig. 8:**
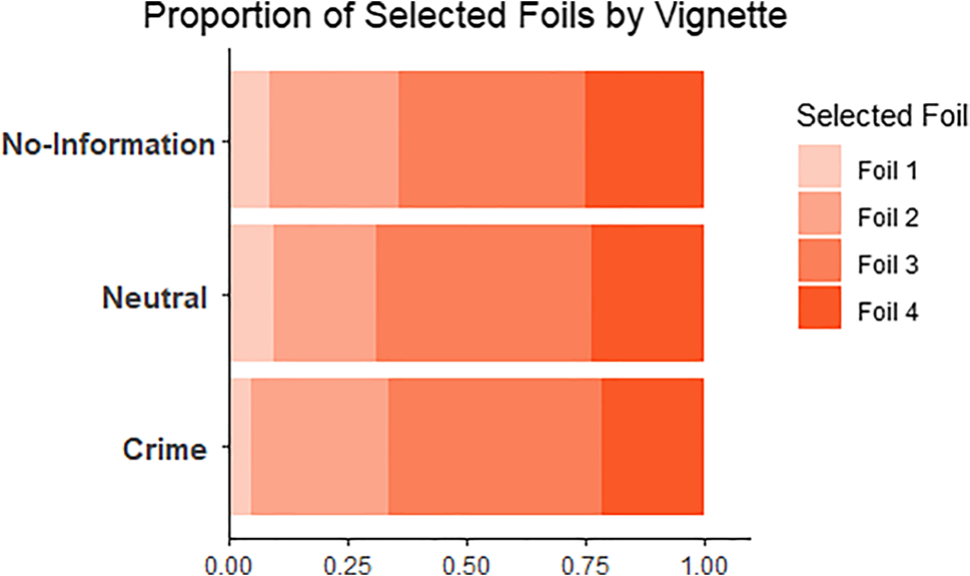
Proportion of foil selections in Experiment 3. Foil 1 represents the least muscular foil, and Foil 4 represents the most muscular foil

This was a target-absent paradigm with no correct response. We found that participants tended to choose more muscular foils than the original target rather than less muscular foils. Participants selected the least muscular foil in 7.5% of cases, and the foil one step below the target level musculature in 25.8% of cases. Conversely, they chose the foil one step above the target level musculature in 43.3% of cases, and the most muscular foil in 23.4% of cases. However, no clear difference emerged between vignette conditions in pattern of response (Fig. 8).

To analyze the potential differences in vignette conditions, we first assessed absolute differences between the three context groups in body selection. To do this, we calculated difference scores for each participant to reflect their chosen body. If the participant had selected the body foil one level above/below the original target musculature, they received a score of +1/−1 that were converted to absolute values to reflect a nondirectional error score. We then used a one-way between-groups ANOVA to investigate differences between the three groups in their errors. Contrary to H1, there were no significant differences between the three context groups, *F*(2, 249) = .664, *p* = .516, partial η^2^ = .005.

To assess whether any of the groups displayed a directional bias in their responses, separate one-sample *t* tests were conducted for each group on the directional error scores, testing whether mean responses deviated significantly from zero. In line with H2, a positive bias was found for those in the crime condition (*M* = .50. *SE* = .14), with participants tending to select foils significantly larger than the original target, *t*(83) = 3.68, *p* < .001, *d =* .40. However, contrary to H2, the same bias was seen in both neutral (*M* = .52, *SE* = .14), *t*(83) = 3.63, *p* < .001, *d* = .40, and no-context conditions (*M* = .45, *SE* = .15), *t*(83) = 3.08, *p* = .003, *d* = .34.

Finally, to assess differences between the groups in this bias, a one-way ANOVA on these directional differences scores was conducted. Contrary to H3, no differences were observed between the three groups, *F*(2, 249) = .065, *p* = .937, partial η^2^ = .001.

### Experiment 3 discussion

In line with our results in Experiments 1 and 2, we found that target bodies presented with criminally charged contexts did not significantly impact participant recall. We found that participants selected significantly larger bodies than the target, but this biased response was common across the three context groups and so cannot be attributable to an activated criminal stereotype. This pattern may reflect Weber’s law (Burt, 1960), whereby our capacity to recognize a change in a stimulus is proportional to the size of the stimulus. In this case, the foil that was least discernible from the original target may have been the one slightly larger than the original, despite the absolute difference in mass between the target and the foil just below the target in musculature being equal to that between the target and the foil just above the target in musculature.

## General discussion

We examined identification accuracy for contextually primed face and body stimuli in three preregistered studies. Despite the predictions of the CMEI, we found no evidence for biased identification of either faces or bodies when paired with criminally charged contexts. Participants viewing images of alleged violent criminals were no more likely to overestimate the facial threat or musculature of the target stimuli than those who studied the targets in empty or neutral contexts. These results suggest that, although errors of eyewitness identification can and do occur, they may not be driven by systematic biases related to how threatening a criminal is later recalled.

Our findings add to the growing skepticism around the field of priming. Conceptual priming broadly refers to the activation of mental concepts via situational cues (Bargh & Chartrand, 2014). This is often through prompted recall of past experience (Callan et al., 2014; Fowler et al., 2011) or as was the case here, prompting participants to think about specific concepts (Cohn et al., 2010; Cohn et al., 2015). This is then used to assess the influence of these primed mental concepts on subsequent behaviours and judgments. Here, we primed participants with the social identity of a target person as a violent criminal, (e.g., Ben-Zeev et al., 2014); however, we found no effect of priming on performance. Our results align with recent failed replications of several prominent priming studies (Yong, 2012). Some suggest that observed priming phenomena are simply the result of false positives and publication bias (Simmons et al., 2011), while others suggest that priming effects are, by their very nature, considerably subtle and thus highly sensitive to even slight variations in experimental methodology (Cesario, 2014). Admittedly, the current study examined the effect of priming on criminal appearance somewhat indirectly, using facial threat and body musculature as proxies of a stereotypically criminal appearance. However, given previous work linking threat and dominance to perceived criminality (Funk et al., 2017; Oosterhof & Todorov, 2008), these measures are useful in exploring the potential role played by context in eyewitness recall. As no effect of priming was observed here across three different studies with varying methodologies, it appears unlikely that priming participants with a criminal context affects their recall of a target’s body morphology or facial threat.

Despite the lack of priming effect and support for the CMEI, this study offers considerable novel advancements in the realm of body recall and identification. Body size is a factor often overlooked as a potential source of prejudice, particularly in legal contexts. Prior investigations have primarily focused on the accuracy of estimation of exact body dimensions, such as height and weight, with these errors often accounting for a high proportion of description errors in culprit recall. However, as suggested by Yuille and Cutshall (1986), we have instead asked participants to *identify* targets based on their body morphology. This study represents the first attempt, to our knowledge, to explore body identification accuracy with careful control over fine morphology changes.

Experiment 1 revealed that, under a simultaneous lineup presentation format, participants were significantly more accurate in identifying briefly presented target bodies when they were high in musculature relative to target bodies low in musculature. This aligns with earlier research that suggests that inherently threatening or negative stimuli are likely to be recalled more accurately than neutral or positive stimuli (e.g., Mattarozzi et al., 2015; Mealey et al., 1996; Oda, 1997; Rule et al., 2012). Thus, it may be in Experiment 1 that the most muscular bodies were recalled with the highest accuracy because they appeared more threatening, consistent with our manipulation check and earlier work (McElvaney et al., 2021).

In Experiment 2, the target body was common across all participants and did not vary in musculature. While not supporting the CMEI, our results support the idea that contextual information can significantly affect person processing (Wieser et al., 2014; Wieser & Brosch, 2012), with participants in the neutral information condition significantly outperforming those in the no-context condition. Although this does not inform whether prosocial or antisocial contexts assist more in later person identification, it does suggest that paired contexts in general may be more helpful that empty (no) contexts.

Taken together, the results from Experiments 1 and uggest that in the absence of contextual information, suggest that in the absence of contextual information, an identification enhancement effect is observed with participants identifying the most threatening (muscular) bodies with the highest level of accuracy. However, when paired with more detailed and salient information, participants are significantly more accurate in identifying target bodies paired with neutral information than bodies not paired with any contextual information. Overall, these findings indicate that memory for body morphology may be enhanced by either the threat-signal of the stimulus or by salient contextual information.

Several limitations should be mentioned in our studies. First, the current methodology lacks ecological validity. Rather than witness a live staged event or video footage of a target culprit committing a staged crime (Ihlebæk et al., 2003; Pozzulo et al., 2008), participants simply read a description of a crime paired with a still target image. Moreover, while a delay was introduced in Experiments 2 and 3, this may still have been too short to reflect the much longer delays of days or weeks between exposure and identification experienced by real eyewitnesses. Given the importance of understanding what factors may affect priming, we sought to increase control at the expense of ecological validity.

In this departure from a true eyewitness procedure, it could be argued that our paradigm was more comparable to a delayed matching task since participants were tasked with identifying the image they had previously studied. It is possible that had a more ecological presentation of criminal target been used, a biased identification may have been observed. Indeed, under real conditions, myriad estimator variables can impinge on later identification. These include the viewing conditions (duration, distance, lighting), distracting stimuli (loud noises, bright lights), and psychological elements (witness motivation, attention), which add uncertainty, leading to a breakdown of accurate sensory communication (Albright, 2017).

Visual uncertainty can play a key role in eyewitness misidentification and increases odds of the intrusion of bias. While some noise was introduced via short target presentation in Experiment 1 and distractor tasks in Experiments 2 and 3 (accuracy in the tasks was lower than in the pilots of pure matching), it is possible that this type of noise was insufficient to activate a bias that would have been amplified by the priming. However, even simple face matching tasks (with no memory component required) are not easy or immune to error (Bruce et al., 1999), In addition, those who excel at unfamiliar face matching tasks tend to also excel at unfamiliar face recognition (Bobak, Dowsett, & Bate, 2016b; Bobak, Hancock, & Bate, 2016a), hinting at important shared mechanisms behind the two processes. Finally, it is unclear that during eyewitness procedures, the witnesses are not trying to do a matching task between the faces in the lineup and the originally viewed face.

Second, regarding our face stimuli, we elected to use tightly controlled CG stimuli that systematically varied in perceived threat due to the close link between facial threat and criminality (Todorov & Oosterhof, 2011). While this provided good experimental control, CG face stimuli can lack the photorealism of real faces (Fan et al., 2012; Fan et al., 2014; Farid & Bravo, 2012), which may disrupt facial identification (Crookes et al., 2015). Despite this, such CG faces have been used extensively and successfully to measure social cognition using behavioural (Brambilla et al., 2021; Oh et al., 2019; Sakuta et al., 2018) and brain imaging methods (Cao et al., 2020; Pelphrey et al., 2003; Todorov et al., 2011). However, the use of CG stimuli may have introduced an additional extraneous confound. Due to the data-driven method by which these faces are created, they may inadvertently correlate with other judgments. In the case of threat, the faces perceived as low in threat may have also been perceived as low in masculinity/high in femininity (Hester et al., 2020). As a result, this may have resulted in easier trials at the lower end of the threat scale than at the higher end, as participants could more reliably reject those more feminine cases as distinct from the original target.

Finally, all stimuli used in this study were Caucasian. Therefore, conclusions drawn from the results are restricted to Caucasian defendants. It is possible that race may interact with body morphology in the perception of threat. For example, Hester and Gray (2018) found that simply being tall increased perceptions of threat for black males only. In addition, we did not record the race of the participants themselves, so there may be an influence of the “other-race” effect (O’Toole et al., 1994). However, we did not test participants on the perceived race of the stimuli, and it is possible that since they were computer generated, participants did not perceive them to be of a particular race.

To address the issues above, future studies could attempt to use morphing software to produce photo-realistic face stimuli (of varying races) that vary in perceived threat. Using a tool such as Psychomorph (Tiddeman et al., 2001) or Abrosoft Fantamorph, two real faces high and low in threat could potentially be morphed together to create a target face of medium threat level (Holtzman, 2011). This morphing process would then allow researchers to create many faces along such a threat continuum, (Holtzman, 2011). This morphing process would then allow researchers to create many faces along such a threat continuum, biasing effects of contextual information on facial memory. Furthermore, a more ecological presentation of the target criminal stimulus, introducing a greater degree of visual uncertainty, with longer delays between target exposure and later recognition, may more accurately test the presence of a bias in criminal recognition.

### Conclusion

We find no evidence of bias in the recollection of targets as a result of contextual framing; neither bodies nor faces were recalled as larger or more threatening than they were. However, we do find that accuracy of body identification is dependent on body morphology. Participant identification accuracy was highest for the most threatening body stimuli high in musculature. In addition, identification improved when the body was paired with detailed neutral contextual information. Limitations notwithstanding, these findings expand the literature on the identification of body morphology. Furthermore, they can help to inform future eyewitness paradigm studies on holistic suspect identification in the wake of a witnessed mock crime.

## Appendix 1: Pilot Studies

The goal of Experiment 1 was to assess the effect of a criminal context on the recollection of presented person stimuli of varying levels of musculature. As these presented stimuli all consisted of CG male bodies generated by the same software, shown in a common stance, it was necessary to add extraneous details to the persons to make it clear that they each represented a different person. To do this, we dressed each of the 10 target person stimuli in varying outfits.

It was thought that the criminal priming context would lead to the culprits being perceived as more threatening than in reality, thus biasing their recollection such that they would be recalled as larger, more muscular than the original target. However, as the predicted effect was to be driven by a perceived threat, it was necessary that the baseline threat (without criminal context) be common across the various outfits. If one of the outfits was inherently more/less threatening than the others, this could lead to a confound in the threat ascribed to the targets. Furthermore, it was necessary to ensure that each of the presented options were visually distinguishable from each other. Should participants have struggled to identify size differences between the options, this may have led to random responding. Therefore, we conducted two pilot studies to address these concerns regarding the threat level of the varying outfits (Pilot 1) and the distinguishability of the presented option stimuli (Pilot 2).

### Pilot 1

As a check on the perceived threat of the various outfits on varying levels of muscular bodies, a pilot study was conducted. Participants were presented with person stimuli of varying levels of musculature, dressed in each of the ten different outfits, and rated them each on perceived threat.

Participants:

Pilot 1 data collection was conducted in November 2019 via the online software Qualtrics, with participants (*N* = 50, 34 female; age: *M* = 42.96 years, *SD* = 12.78) recruited from Prolific, an online crowdsourcing platform.

Stimuli (Bodies):

We manipulated our model body’s appearance by applying increments on Daz’s musculature scale to an initial Standard body stimulus (Genesis 8 Basic Male). This Standard body is initially set to 0% on the scale. This scale was then applied to the Standard body in increments of 50%. This resulted in stimuli that varied in three levels of musculature: 0%, 50% and 100%.

Ten different outfits were compiled and added to these three base bodies using the “Genesis 8 MEGA Wardrobe” package in Daz Studio. This package gives a range of various clothing items with which to dress the model, as well as the option of changing the colours of the items. This gave a total of 30 different body stimuli.

Procedure:

This experiment followed a within-participant design. All participants rated each of the 30 different body stimuli presented in a randomised order. Participants completed one blocks of stimuli ratings, in which they viewed the 30 body stimuli and were asked to rate the threat of the stimuli on a scale from 1 (“Not At All Threatening”) to 7 (“Extremely Threatening”).

Results:

Scores for each outfit’s perceived threat was averaged across the three presented levels of musculature. As the scores for some of the outfits fell beyond the bounds of normality, we used a Friedman test to assess the effect of outfit on perceived threat. This showed that perceived threat varied significantly across the outfits, *X*^*2*^ (9) = 39.71, *p* < .001.

Post-hoc examination was carried out using Wisconsin Signed-Rank tests to compare each set of outfits, with a Bonferroni correction applied to account for multiple comparisons. These indicated that significant differences arose for outfits 4, 7 and 9. Specifically, outfit 4 tended to be perceived as more threatening than the others, while 7 and 9 were perceived as less threatening.

On inspection, these outlying outfits differed from the rest of the clothing in clear ways. Specifically, outfit 4 was the only one to include a tank top, which most likely led to it being seen as more threatening. In contrast, outfits 7 and 9 were the only ones that included shorts, which probably led to their perception as less threatening. These divergences were thus fixed for the final outfit stimuli used in Experiment 1.

## Pilot 2

To ensure that participants could distinguish between the stimuli, they were asked to select a body out of choice of 5 bodies of varying musculature that matched the target. This matched the set-up of the procedure undertaken by those in the no context condition of Experiment 1, with the sole difference being that the target stimulus remained on-screen at all times. Hence this was simply a matching task.

Participants:

Pilot 2 data collection was conducted in March 2020. A convenience sample of 6 participants completed the task (2 female, age: *M* = 24.50 years, *SD* = 1.76).

Procedure:

As in Experiment 1, participants initially completed two trials at the lowest level of musculature (0%), two at the highest level (100%) and six at the central level (50%). A target body stimulus was presented on screen, below which were five optional body stimuli varying in musculature. On each trial they had to select the option that matched the target body. No time limit was imposed, the target and options remained on screen until participants had made their selection.

We first examined musculature differences of 20% between the options. For example, with a target body of 50% musculature, options were presented (in a randomised order) at 10%, 30%, 50%, 70% and 90% musculature. We found that these musculature differences of 20% between the body options led to an average performance accuracy across all 10 trials of 81.67%.

We then repeated the pilot with musculature differences of 25% between the options. For example, with a target body of 50% musculature, options were presented at 0%, 25%, 50%, 75% and 100% musculature. This led to a performance accuracy of 100%. We therefore used a step size of 25% in the main experiment.

## Appendix 2: Experiment 2 Confidence Results

Regarding confidence measures, we expected that participants would show greater confidence on easier foil trials than difficult foil trials (H7), and that more confident participants would display greater accuracy than less confident participants (H8) across all conditions.

*Face Confidence*

As with accuracy, confidence was calculated for each threat level by averaging the participant’s confidence rating across each of the four trials completed at that level. We used mixed ANOVAs to assess the effects of context (between-subject) and threat level (within-subject) on participant confidence. We found a significant effect of threat level on confidence, *F*(5.54, 696.73) = 20.59, *p* < .001, partial η2 = .095. Consistent with H7, confidence was highest on -2SD threat (*M* = 81.98, *SE* = 1.23), which corresponded to the test level with the highest accuracy. Confidence on these trials was significantly (*p* < .001) higher than all other threat levels, followed by +2SD (*M* = 75.42, *SE* = 1.40), +1SD (*M* = 72.87, *SE* = 1.60), -1SD (*M* = 71.77, *SE* = 1.34) and 0SD (*M* = 70.89, *SE* = 1.27). We also found significant differences between confidence on the 0SD trials and +2SD trials (*p* = .005).

There was no main effect of context, nor a significant interaction between context and threat level. Finally, contrary to H8, there was no correlation between mean participant accuracy and mean participant confidence across trials, *r* = .11, *p* = .138.

*Body Confidence*

As with the faces, we used mixed ANOVAs to assess the effects of context (between-subject) and test musculature (within-subject) on participant confidence. We found a significant effect of musculature on confidence, *F*(5.51, 690.43) = 10.11, *p* < .001, partial η2 = .049. Consistent with H7, confidence was highest on the Level 6 Muscle trials (*M* = 72.30, *SE* = 1.37) and Level 2 Muscle trials (*M* = 70.61, *SE* = 1.35). Participants were significantly (*p* < .001) more confident on the Level 6 muscle trials than on Level 5 (*M* = 68.07, *SE* = 1.30), Level 4 (target) (*M* = 68.17, *SE* = 1.34) and Level 3 (*M* = 66.84, *SE* = 1.39) trials, with confidence on Level 2 also significantly higher (*p* < .001) than on Level 3.

There was no main effect of context, nor a significant interaction between context and threat level. Finally, contrary to H8, we found no correlation between mean participant accuracy and mean participant confidence across trials, *r* = -.13, *p* = .068.

## Appendix 3: Crime/Neutral Context Vignettes

### Crime Vignette 1 (adapted from Porter et al. (2010))

In August 2019, a corner shop was robbed in a London suburb. At the time the only people in the shop were the shopkeeper behind the counter, and one customer who was shopping in the back, who hid when the culprit entered.

The culprit threatened the shopkeeper with what appeared to be a large kitchen knife. After the shopkeeper handed over £140 from the cash register, the culprit demanded more money. When the shopkeeper failed to provide the additional money, the culprit stabbed him once in the chest, and then ran out. Unfortunately, emergency medical teams were unable to save the shopkeeper.

### Crime Vignette 2 (adapted from Mendelsohn and Sewell (2004))

In October 2019, a man was walking to his car after running some errands in central London. He was approached by a stranger who began to verbally insult him. The man walked quickly toward a busy intersection, but the stranger caught up with him.

The stranger pulled out a knife and pushed the man into a deserted alley. The stranger held the knife to the man’s throat and threatened to kill him if he did not hand over his wallet and watch. After grabbing his possessions, the stranger pushed the man to the ground and stabbed him in the side. The stranger then ran off, leaving the man on the ground. Unfortunately, emergency medical teams were unable to save the man.

### Neutral Vignette 1

In August 2019, a corner shop in a London suburb sold a winning lottery scratchcard to one of their customers. At the time of sale the only people in the shop were the shopkeeper behind the counter, one customer who was shopping in the back and the eventual lottery winning customer.

The winning customer purchased the lottery scratchcard from the shopkeeper with their debit card. After the shopkeeper handed over the winning ticket, the customer immediately scratched the card. Upon seeing that they had won a large cash-prize, the customer ran out of the shop. Unfortunately, they had left their debit card on the counter of the shop.

### Neutral Vignette 2

In October 2019, a man was walking to his car after running some errands in central London. He was approached from a distance by a stranger who called out to him to stop. The stranger caught up to the man by a busy intersection.

The stranger pulled out a mobile phone and showed it to the man. The stranger indicated that they were lost, held the phone to the man’s face and showed him an address on the screen. The displayed destination was approximately a 20 minute walk east from the intersection. After the man pointed the stranger in the right direction, the stranger ran off while thanking the man. Unfortunately, their debit card had fallen out of their pocket as they had taken out their phone.

## Footnotes

1 For the analysis of absolute difference scores, participants lying outside of three standard deviations from the mean were excluded as outliers. For both face and difference scores, 10 of the 220 participants were excluded.
